# RNA-seq and Ribosome Profiling Reveal the Translational Landscape of Rice in Response to Rice Stripe Virus Infection

**DOI:** 10.3390/v16121866

**Published:** 2024-11-29

**Authors:** Chen Wang, Yao Tang, Changmei Zhou, Shanshan Li, Jianping Chen, Zongtao Sun

**Affiliations:** 1 College of Plant Protection, Nanjing Agricultural University, Nanjing 210095, China; wangchen199766@163.com (C.W.); ty1049784783@163.com (Y.T.); zhouchangmei11@163.com (C.Z.); lishanshan106@163.com (S.L.); 2 State Key Laboratory for Managing Biotic and Chemical Threats to the Quality and Safety of Agro-Products, Key Laboratory of Biotechnology in Plant Protection of MARA, Key Laboratory of Green Plant Protection of Zhejiang Province, Institute of Plant Virology, Ningbo University, Ningbo 315211, China

**Keywords:** ribosome profiling, RNA-seq, rice stripe virus, translational efficiency, uORFs

## Abstract

Rice is a crucial staple food for over half the global population, and viral infections pose significant threats to rice yields. This study focuses on the Rice Stripe Virus (RSV), which is known to drastically reduce rice productivity. We employed RNA-seq and ribosome profiling to analyze the transcriptional and translational responses of RSV-infected rice seedlings. Our results reveal that translational reprogramming is a critical aspect of the plant’s defense mechanism, operating independently of transcriptional changes. Notably, less than half of the differentially expressed genes showed concordance between transcription and translation. Furthermore, RSV infection led to significant alterations in translational efficiency for numerous genes, suggesting that the virus selectively manipulates translation to enhance its pathogenicity. Our findings underscore the necessity of examining both transcriptional and translational landscapes to fully understand plant responses to viral infections.

## 1. Introduction

Rice (*Oryza sativa* L.) is a staple food crop in many Asian countries, including China, India, Indonesia, Japan, Korea, and the Philippines, with approximately 50% of the global population relying on it for sustenance. The demand for rice continues to rise significantly across various regions [1,2]. However, rice viruses pose a serious threat to crop yields, leading to symptoms such as dwarfism, altered tillering, wilting, and yellowing, all of which adversely affect production [3]. One particularly destructive virus is the Rice Stripe Virus (RSV), a member of the *Tenuivirus* genus, known for its severe impact on rice yields [4]. RSV can cause curling and discontinuous yellow stripes on leaves and even give rise to enormous losses in field production, especially in East Asia [5,6]. RSV is transmitted by the small brown planthopper (SBPH) in a persistent circulative–propagative manner [7]. Infected rice plants typically exhibit chlorosis, weakness, and necrosis in new leaves, resulting in stunted growth and substantial yield losses [8,9,10]. Thus, a thorough investigation into the mechanisms of RSV infection is crucial for developing effective antiviral strategies and enhancing disease resistance through targeted engineering [11].

Recent advancements in high-throughput methodologies, especially RNA sequencing (RNA-seq), have revolutionized gene expression studies [12]. Several developments in RNA-seq methods have provided an even more complete characterization of RNA transcripts and solved many problems in plants [9,13,14]. However, mRNA levels do not always accurately reflect protein production, as gene expression is regulated at both transcriptional and translational levels through mechanisms such as microRNAs and ribosome stalling [15,16,17]. Consequently, there is a critical need for techniques that directly monitor protein synthesis [18,19,20]. A novel method known as ribosome profiling has recently been developed, allowing for single-nucleotide-resolution measurement of protein synthesis by deep sequencing ribosome-protected mRNA fragments (RPFs) [18,21,22]. As a significant methodological advancement, ribosome profiling has rapidly been applied to understand the translational landscape in various species [18,23,24]. In plants, this technique has been employed to analyze responses to drought and salt stress in rice [25,26]. Evidence increasingly suggests that transcriptional and translational responses to stress are relatively independent processes [27,28,29,30]. However, there is still limited research applying ribosome profiling to investigate translation regulation following viral infection.

In this study, we conducted RNA-seq and ribosome profiling on healthy and RSV-infected seedlings to comprehensively understand molecular responses to viral infection. Our analysis revealed significant differences in transcriptomic and translatomic profiles between the two groups, suggesting that translational reprogramming constitutes a distinct layer of the plant’s response to RSV infection that is independent of transcriptional changes. Fewer than half of the differentially expressed genes (DEGs) were common to both transcription and translation, with many genes exhibiting altered translational efficiencies post-infection. This indicates that the virus may selectively modulate the translation of specific mRNAs to enhance its pathogenicity. Overall, our study advances understanding of the molecular basis of RSV infection and emphasizes the importance of examining both transcriptional and translational responses to fully appreciate the plant’s adaptive mechanisms against viral threats. Future investigations could focus on identifying regulatory elements governing these translational changes and exploring their potential applications in improving disease resistance in rice and other crops.

## 2. Result

### 2.1. Overview of RNA-seq and Ribo-Profiling

**Figure 1:**
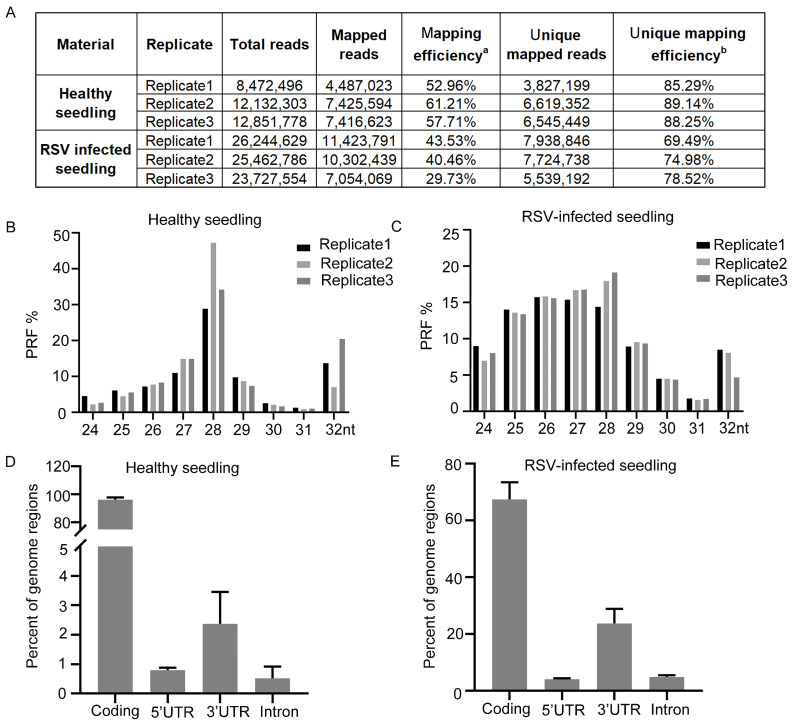
The characteristics of ribosome profiling data for healthy and RSV-infected rice seedlings. (**A**) Alignment statistics for ribosome profiling reads. Mapping efficiency is defined as the total number of mapped reads divided by the total number of reads. Unique mapping efficiency represents the proportion of unique reads among the total mapped reads. (**B**) Length distribution of ribosome protected fragments (RPFs) in healthy seedlings. The grey, black, and white bars correspond to three biological replicates. (**C**) Length distribution of ribosome protected fragments (RPFs) in RSV-infected rice seedlings. The grey, black, and white bars correspond to three biological replicates. (**D**) The percentages of RPF reads located in CDSs, 5′ UTRs, and 3′ UTRs in healthy seedlings. (**E**) The percentages of RPF reads located in CDSs, 5′ UTRs, and 3′ UTRs in RSV-infected rice seedlings.

**Figure 2:**
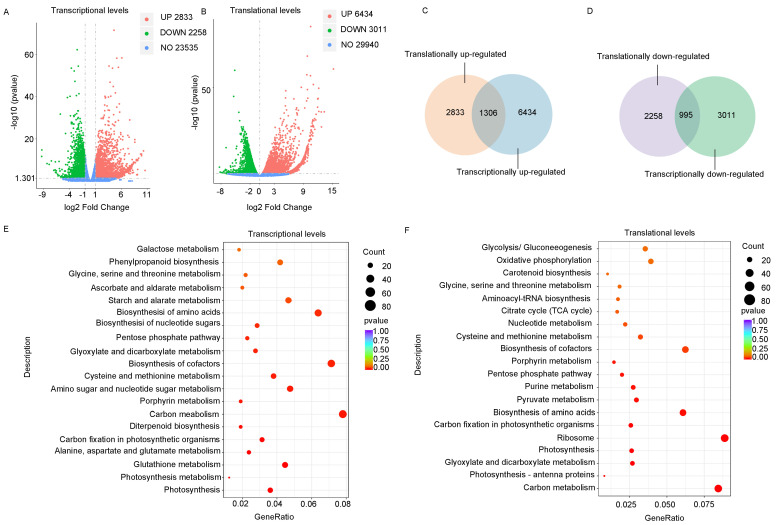
RSV infection-induced transcriptional and translational responses. (**A**) The number of differentially expressed genes (DEGs) at transcriptional levels. Genes with a fold change ≥ 2 and a q-value ≤ 0.05 are displayed. The horizontal axis shows the log2 fold changes, and the vertical axis represents the −log10 *p*-values. The blue dashed line indicates the threshold for differential gene screening. (**B**) The number of differentially expressed genes (DEGs) (fold change ≥ 2 and q-value ≤ 0.05) at translational levels. The representation is similar to that of panel A, with the same axis representations. (**C**) The relationship between upregulated genes at transcriptional and translational levels. (**D**) The relationship between downregulated genes at transcriptional and translational levels. (**E**) KEGG (Kyoto Encyclopedia of Genes and Genomes) pathway enrichment analysis at transcriptional levels, using a *p*-value < 0.05 as the threshold for significant enrichment. (**F**) KEGG (Kyoto Encyclopedia of Genes and Genomes) pathway enrichment analysis at translational levels, using a *p*-value < 0.05 as the threshold for significant enrichment. Gene ratios represents the ratios of DEGs annotated with KEGG pathways to the total number of DEGs, with a *p*-value < 0.05 as the threshold for significance.

To systematically characterize the transcriptional and translational responses during virus infection at a genome-wide scale, we conducted RNA-seq and ribosome profiling on both healthy and RSV-infected rice seedlings, each with three biological replicates. Compared to healthy plants, samples infected with RSV exhibit obvious symptoms and higher levels of viral RNA (Figure S1A). On average, we generated approximately 11 million ribosome profiling reads for healthy seedlings and 25 million for those infected with RSV (Figure 1A). During the translation process, ribosomes move relative to RNA in units of codon length (3 nt) [31,32,33,34]. And we observed a strong three-nucleotide periodicity when scanning after the start codon and before the stop codon in both healthy and RSV-infected seedlings (Figures S2A and S3A). After initial processing to filter out rRNA, low-quality, and tRNA reads, about 30% to 60% of the total reads mapped to the *Oryza sativa* Japonica Group reference genome (RefSeq: GCF_034140825.1) (Figure 1A). To investigate the impact of virus infection on ribosome profiling characteristics, we compared data between healthy and RSV-infected seedlings. We observed that the length of ribosome footprints (RPFs) in healthy samples is around 28 nt, with a distribution of 25–28 nt and a peak at 28 nt in RSV-infected samples (Figure 1B,C). In healthy seedlings, 0.79% and 2.3% of RPFs were found in 5′ UTRs and 3′ UTRs, respectively, while 96.3% were located in coding sequences (CDSs) and 0.52% in introns (Figure 2D). In RSV-infected seedlings, 0.4% and 23.7% of RPFs were found in 5′ UTRs and 3′ UTRs, respectively, with 67.3% in CDSs and 4.8% in introns (Figure 2E). These changes imply that translation in UTRs may mediate the plant response to RSV infection.

### 2.2. Virus Infection Altered Expression at Both Transcriptional and Translational Levels

Using both RNA-seq and ribosome profiling data of healthy and RSV-infected plants, we were able to examine the effects of virus infection on gene expression at both transcriptional and translational levels simultaneously. Based on the criteria of an absolute fold change ≥ 2 and a *p*-value < 0.05, we obtained 2833 upregulated and 2258 downregulated DEGs (Figure 2A). Meanwhile, 6434 upregulated genes and 3011 downregulated genes were detected at the translational levels (Figure 2B). The numbers of upregulated genes and downregulated genes at transcriptional levels were lower than those at translational levels, indicating that viral infection can cause a significant amount of gene regulation at translational levels. We found that only 1306 DEGs overlapped in genes with upregulated transcription and translation levels (Figure 2C) and that only 996 DEGs overlapped in genes with downregulated transcription and translation levels (Figure 2D), accounting for 20% and 33% of all translational DEGs, respectively. Notably, DEGs at both levels were enriched in amino acid biosynthesis pathways, highlighting that viral infection triggers extensive protein synthesis and metabolic reactions (Figure 2E,F). The differences in the number and pathways of DEGs at both levels emphasize that gene regulation occurs at both transcriptional and translational stages post-infection, rather than solely at the transcriptional level.

### 2.3. RSV Infection Introduced Significant Changes in the Translational Efficiencies of a Large Number of Genes

**Figure 3:**
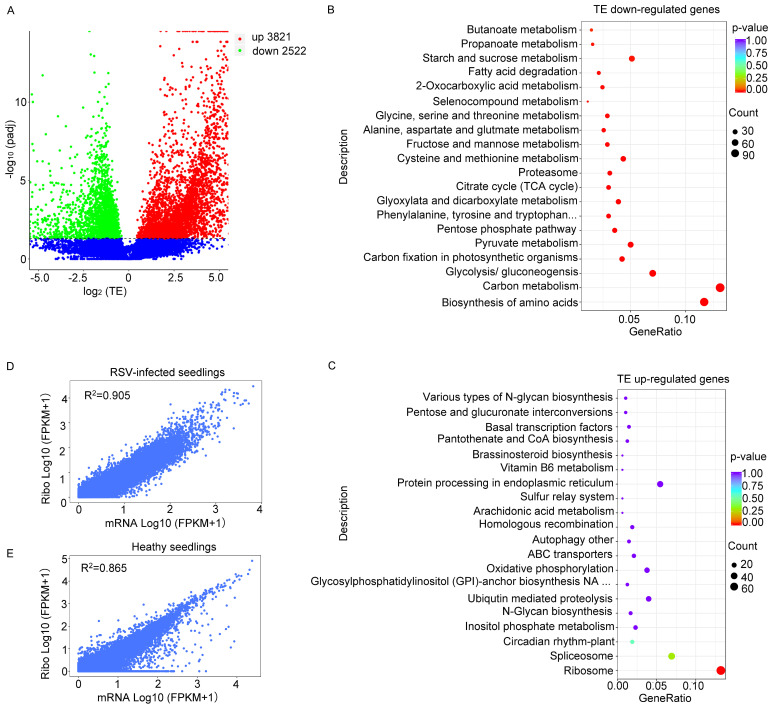
RSV infection-induced transcriptional and translational responses. (**A**) The number of TEs (fold change ≥ 2 and q-value ≤ 0.05) at the transcriptional level. TEs with a fold change ≥ 2 and a q-value ≤ 0.05 are displayed. The horizontal axis shows the log2 fold changes, and the vertical axis represents the −log10 *p*-values. (**B**) KEGG (Kyoto Encyclopedia of Genes and Genomes) pathway enrichment analysis at transcriptional levels, using a *p*-value < 0.05 as the threshold for significant enrichment. (**C**) KEGG (Kyoto Encyclopedia of Genes and Genomes) pathway enrichment analysis at transcriptional levels, using a *p*-value < 0.05 as the threshold for significant enrichment. (**D**) Pearson correlation coefficients between the ribo-profiling level and RNA-seq in RSV-infected seedlings. (**E**) Pearson correlation coefficients between the ribo-profiling level and RNA-seq in healthy seedlings.

Translational efficiency (TE; calculated by RPKM in Ribo-Profiling/RPKM in RNA-seq) serves as a key indicator of RNA utilization efficiency [18]. We assessed how rice seedlings respond to RSV infection through alterations in TE. A total of 3821 genes exhibited increased TE, while 2522 genes showed decreased TE under RSV infection compared to the healthy seedling controls (Figure 3A). Overall, the downregulated TE genes were predominantly enriched in pathways related to amino acid biosynthesis and carbon metabolism (Figure 3B). The upregulated TE genes were primarily associated with pathways involving spliceosome and ribosome biosynthesis (Figure 3C). In order to analyze the relationship between transcription and translation levels, we calculated the Pearson correlation coefficients for the transcriptome and translation groups. Under RSV-infected conditions, the Pearson correlation coefficient between gene transcription and translation levels was 0.905 (Figure 3D). And the Pearson correlation coefficient between gene transcription and translation levels in healthy seedlings was 0.865 (Figure 3E), indicating a strong synergistic effect between transcription and translation under stress conditions. These findings suggest that rice plants may employ independent translational regulation in response to RSV infection.

### 2.4. RSV Infection Altered Gene Translation Efficiency 

**Figure 4:**
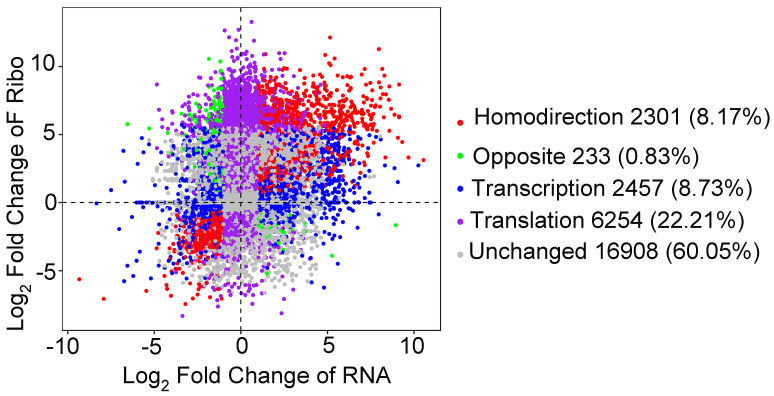
Fold changes at the transcription and translation levels with RSV infection. Scatter plot of fold changes at the transcription and translation levels of RSV-infected vs. healthy seedlings. Genes were classified into five distinct groups based on their expression changes, defined by fold-change values ≥ 2 and false-discovery rates (FDRs) below 0.05.

To understand the effect of RSV infection on transcription and translation levels in more detail, the genes that changed markedly at the transcription or translation level were defined by the criteria of fold-change values ≥ 2 and false-discovery rates (FDRs) below 0.05. We subdivided all genes into five groups (Figure 4). A homodirectional group (8.17%, 2301 genes) changed markedly at both the transcriptional and translational levels and showed consistent trends. An opposite group (0.83%, 233 genes) changed significantly at both the transcriptional and translational levels and showed an inconsistent trend. A transcription group (8.73%, 2457 genes) changed markedly only at the transcriptional level. A translation group (22.21%, 6254 genes) changed markedly only at the translational level. An unchanged group (60.05%, 16908 genes) did not change markedly at either the transcriptional or the transactional level. Except for the unchanged group, only 8.17% of genes exhibited the same trend at the transcription and translation levels, indicating that for some genes there is independence between transcriptional and translational regulation. And only a few genes showed the opposite trend; so, for the regulation of most genes, the translation level cannot override the transcription level.

### 2.5. Functional Comparison at the Transcription and Translation Levels

**Figure 5:**
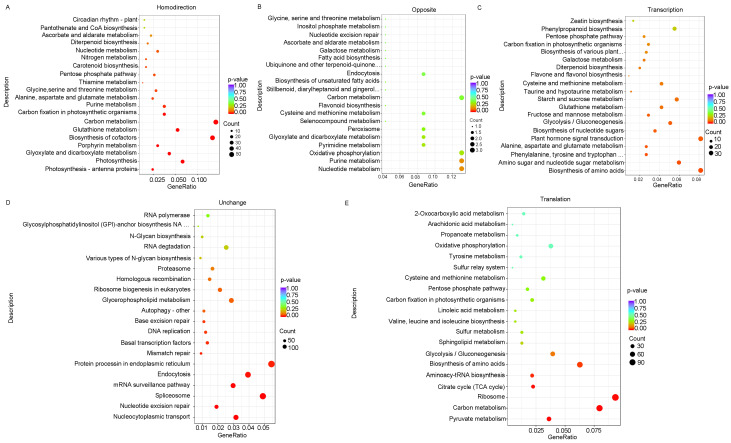
Top 20 KEGG pathways of DEGs at transcriptional and translational levels. (**A**) KEGG pathway analysis of genes in the homodirectional group. (**B**) KEGG pathway analysis of genes in the opposite group. (**C**) KEGG pathway analysis of genes in the transcription group. (**D**) KEGG pathway analysis of genes in the unchanged group. (**E**) KEGG pathway analysis of genes in the translation group.

We further performed a pathway enrichment analysis on the genes in the five groups. Genes in the homodirectional group were significantly enriched in carbon metabolism, biosynthesis of cofactors, and several pathways related to photosynthesis, such as photosynthesis—antenna proteins, photosynthesis, and circadian rhythm—plant (Figure 5A). Few genes showed the opposite trend at transcription and translation levels. Genes in the opposite group were enriched in oxidative phosphorylation, purine metabolism, and nucleotide metabolism (Figure 5B). Genes in the transcription group were significantly enriched in plant hormone signal transduction, biosynthesis of amino acids, and amino sugar and nucleotide sugar metabolism pathways (Figure 5C). Amino acids are precursors for the synthesis of many metabolites and have multiple functions in the growth and other biological processes of organisms [35]. Plant hormones play pivotal roles in the regulation of immune responses to microbial pathogens, insect herbivores, and beneficial microbes [36], which are significantly regulated at the transcriptional level under viral infection. Genes in the unchanged group were enriched in protein processes, endocytosis, nucleocytoplasmic transport, and spliceosomes (Figure 5D). Genes in the translation group were enriched in ribosomes, carbon metabolism, biosynthesis of amino acid pathways, and glycolysis/gluconeogenesis (Figure 5E). 

### 2.6. Identification and Characteristics of Upstream Open Reading Frames (uORFs)

**Figure 6:**
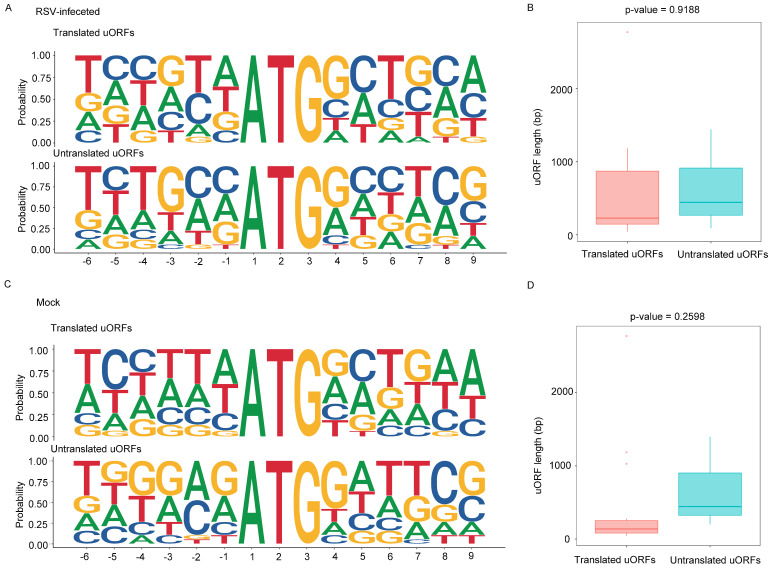
Characteristics of uORFs in healthy seedlings and RSV-infected seedlings. (**A**) Sequence composition of translated (top) and untranslated uORFs (lower) around the ATG start codon in the RSV-infected seedlings. (**B**) Comparison of translated and untranslated uORFs by uORF length in the RSV-infected seedlings. (**C**) Sequence composition of translated (top) and untranslated uORFs (lower) around the ATG start codon in the healthy seedlings. (**D**) Comparison of translated and untranslated uORFs by uORF length in the healthy seedlings.

Upstream open reading frames (uORFs)—short coding sequences located 5′ of the main coding sequence—are found in a minority of eukaryotic mRNAs and often play regulatory roles [37]. These uORFs precede the translation start site for downstream main open reading frames (mORFs) [38,39]. Utilizing Ribo-seq data, we conducted a genome-wide identification of uORFs based on the ATG start codon located in the 5′ untranslated region (UTR). We first performed a motif analysis to examine the frequency of nucleotides surrounding the uORF start codon in both translated and untranslated regions, as this codon is crucial for translation initiation. Our analysis revealed that the probability of cytosine at the −1 and −2 positions was higher in untranslated uORFs compared to translated uORFs in RSV-infected seedlings (Figure 6A, Supplementary Table S1). Additionally, in healthy seedlings, the probability of guanine at the −1 position was also higher in untranslated uORFs than in translated uORFs, with a greater overall frequency of guanine in the untranslated uORFs (Figure 6C, Supplementary Table S2). To compare the characteristics of translated and untranslated uORFs, we examined their lengths. We found that the lengths of translated uORFs were shorter than those of untranslated uORFs in healthy seedlings (Figure 6D). However, this difference diminished under RSV infection (Figure 6B). These findings indicate that the translation of uORFs in rice plants is regulated in response to RSV infection, suggesting a complex interplay between translation and viral response mechanisms. 

## 3. Discussion

*Rice Stripe Virus* (RSV) has become a major pathogen of rice [40]. Over the past few decades, numerous studies have utilized RNA sequencing technology to generate high-throughput sequence data [28,41]. These findings indicate that the downregulation of genes associated with photosynthesis and flowering is closely linked to the disease symptoms induced by RSV [42]. Early in the infection process, RSV triggers the expression of many host defense genes; however, in the later stages, the majority of these upregulated defense genes are suppressed [41]. It is important to note that mRNA levels do not always accurately reflect the regulation of protein translation [43,44]. Ribosome profiling, which maps the positions of millions of translating ribosomes within the cell, allows for a genome-wide investigation of translation [18,19,45]. We employed this technique to examine translation during RSV infection. By successfully obtaining both ribosome profiling and RNA-seq data, we were able to assess genome-wide gene expression changes at the translational level post-RSV infection and explore the interactions between transcriptional and translational responses. 

Overall, we observed a strong correlation between the fold changes in gene expression at both the transcriptional and translational levels, regardless of RSV infection (Figure 3D,E). Notably, there were more differentially expressed genes at the translational level compared to the transcriptional level following RSV infection. There were 6434 upregulated genes and 3011 downregulated genes at the translation level, while there were only 2833 upregulated genes and 2258 downregulated genes at the transcription level, indicating that a large number of genes were intentionally regulated at the translation level (Figure 2A,B). Ribosome profiling quantifies the genome-wide ribosome occupancy of transcripts. By integrating this data with matched RNA sequencing, we can calculate the TEs of genes to reveal translational regulation [46,47]. After RSV infection, we observed that 3821 genes were upregulated and 2522 were downregulated in terms of TE, highlighting the specific regulation of translation in plants. Additionally, the changes in translated and untranslated uORFs following RSV infection differed, suggesting that uORFs also play a role in plant antiviral regulation.

In this article, we enhanced the analysis of differentially expressed genes at both the transcriptional and translational levels post-RSV infection. Our findings reveal that certain genes are specifically regulated at the translational level after viral infection, which will aid in our understanding of the mechanisms of virus infection and inform strategies to protect rice crops from viral diseases.

## 4. Methods

### 4.1. Plant Materials and Growth Conditions

The rice cultivar ZH11 was used as the wildtype variety in this study. Seeds were germinated by soaking them in water in a 36 °C incubator for three days. After germination, the seedlings were transferred to a greenhouse maintained at 28–30 °C with a 14/10 h light/dark cycle.

### 4.2. Insect Vectors and Virus Infection

The transmission of RSV was facilitated by the small brown planthopper (*Laodelphax striatellus*). For the virus transmission experiments on transgenic rice, virus-free nymphs were fed on RSV-infected rice plants for 3–5 days. The insects were then moved to healthy rice seedlings for approximately 10 days to allow for viral circulation within the insects. Subsequently, 2–3 SBPHs carrying RSV were placed on each transgenic rice plant at the 3- to 4-leaf stages for a duration of 3 days. Concurrently, the same number of virus-free insects was introduced to another set of rice seedlings as a negative control. After the feeding period, all insects were removed, and the inoculated plants were grown in a fertile field to monitor for symptoms [9,10]. The primers used in the qPCR analysis are listed in Supplementary Table S3.

### 4.3. Ribosome Profiling

To digest RNA other than RPFs, cell or tissue lysates were treated with the non-specific endoribonuclease RNase I. Size-exclusion chromatography was performed using a MicroSpin S-400 HR column to separate monosomes. RPF fragments (20–38 nt) were purified via PAGE gel. Subsequently, the ends of these fragments were phosphorylated and ligated to 5′- and 3′-end adapters. The RNA samples were then processed with an rRNA depletion kit (Qiagen, 334387) to minimize rRNA contamination. Following this, the fragments underwent reverse transcription to generate cDNAs, which were then amplified by PCR [48,49]. After constructing the library using the Multiplex Small RNA Library Prep Set for Illumina (Set1, NEB, E7300L), the concentration of the library was measured with the Qubit^®^ 2.0 Fluorometer and adjusted to 1 ng/μL. The insert size of the library was assessed using the Agilent 2100 Bioanalyzer. Finally, the precise concentration of the cDNA library was verified using qPCR. Once the insert size and concentration were confirmed to be correct, the samples were ready for sequencing.

### 4.4. mRNA Sequencing Library Construction

Total RNA was extracted using TRIzol reagent (Invitrogen, Carlsbad, CA, USA), following the manufacturer’s instructions. The HISAT2 v2.0.5 was used to build the index for the reference genome, and paired-end clean reads were aligned to the reference genome using HISAT2 v2.0.5. We chose HISAT2 as the alignment tool.

### 4.5. Quality Control for Raw Data

The raw FASTQ data were processed using internal Perl and Python scripts. In this step, clean reads were obtained by removing the following reads: reads with 5′ adapter sequences; reads lacking 3′ adapter sequences or insertion sequences; reads with more than 10% ambiguous bases (N); reads for which over 30% of bases had a Qphred score ≤ 20. The 3′ adapter sequences of the reads were trimmed, and the Q20, Q30, and GC contents of the clean data were calculated. Next, Bowtie (version 1.1.2) was used to align the reads with rRNA sequences from the SILVA database, identifying and discarding reads that aligned to rRNA without allowing mismatches. Bowtie was also used to align the reads with tRNA sequences from the Rfam database, identifying and discarding the remaining reads derived from tRNA, again without allowing mismatches [50]. Subsequent analyses were conducted based on high-quality clean data.

### 4.6. Quantification of Gene Expression Levels

Quantification of mapped results at the gene level was carried out using HTSeq (0.9.1). HTSeq is a Python package that calculates the number of mapped reads for each gene. RPKM values were generated to represent the gene expression level of each specific gene. 

### 4.7. Differential Expression Analysis

Differential expression analysis was performed using the DESeq2 R package (version 1.14.1). DESeq2 is a statistical analysis tool based on a negative binomial distribution model that identifies differential expression in digitized gene expression data. The Benjamini–Hochberg procedure was used to control the false-discovery rate and adjust the resulting *p*-values. The significance criteria for differentially expressed genes were set as follows: |log2(Fold Change)| > 0 and *p*-value < 0.05.

### 4.8. uORF Analysis

uORF expression was assessed using an RPKM threshold ≥ 1 [26]. Subsequently, the R library (SeqLogo) was utilized to visualize the enrichment of motifs surrounding the start codons of expressed versus non-expressed uORFs.

### 4.9. TE Analysis

TE was calculated as the ratio of RPKM values from Ribo-seq to RPKM values from RNA-seq. For samples with biological replicates, differential TE analysis was performed using RiboDiff (version 0.2.1) [51,52].

## Data Availability

Any information required to reanalyze the data reported in this paper will be shared by the lead contact upon reasonable request.

## Supplementary Materials

The following supporting information can be downloaded at: https://www.mdpi.com/article/10.3390/v16121866/s1, Figure S1: Healthy rice seedlings and RSV infected rice seedlings (A) Symptoms in rice plants and WT after RSV-infection. (B) RT-qPCR analysis of the relative mRNA levels of RSV CP in RSV-infected rice plants and WT ZH11 at 30 dpi. Figure S2: (A) Three-nucleotide periodicity in heathy seedlings. Figure S3: (A) Three-nucleotide periodicity in RSV-infected seedlings. Table S1: uORFs analysis in RSV-infected seedings. Table S2: uORFs analysis in healthy seedings. Table S3: The primers using to viral RNA analysis.
