# Supplementary material for: RNA-seq and Ribosome Profiling Reveal the Translational Landscape of Rice in Response to Rice Stripe Virus Infection
Source: Viruses. 2024 Nov 29;16(12):1866. doi: 10.3390/v16121866 (PMC11680141; doi:10.3390/v16121866)
Supplement: Supplementary file 1 [file viruses-16-01866-s001.zip › Supplementary Fig.pdf]

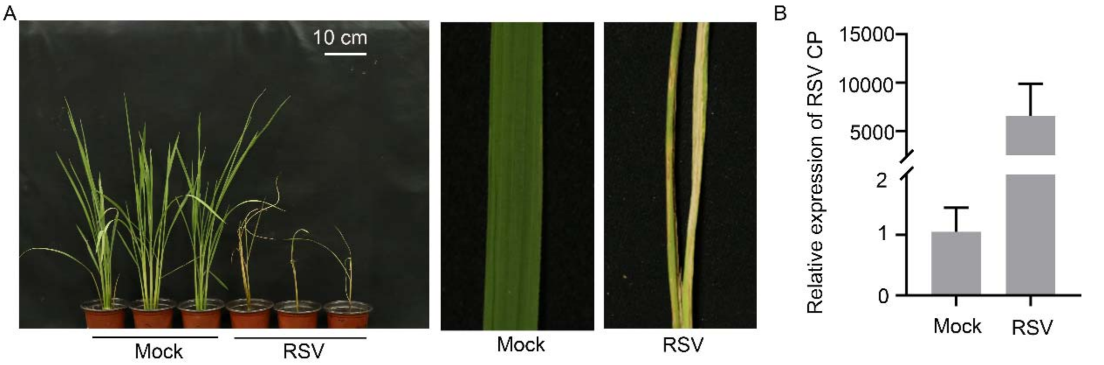

Supplementary Figure S1 | Healthy rice seedlings and RSV infected rice seedlings

(A) Symptoms in rice plants and WT after RSV-infection.

(B) RT-qPCR analysis of the relative mRNA levels of RSV CP in RSV-infected rice plants and WT ZH11 at 30 dpi.

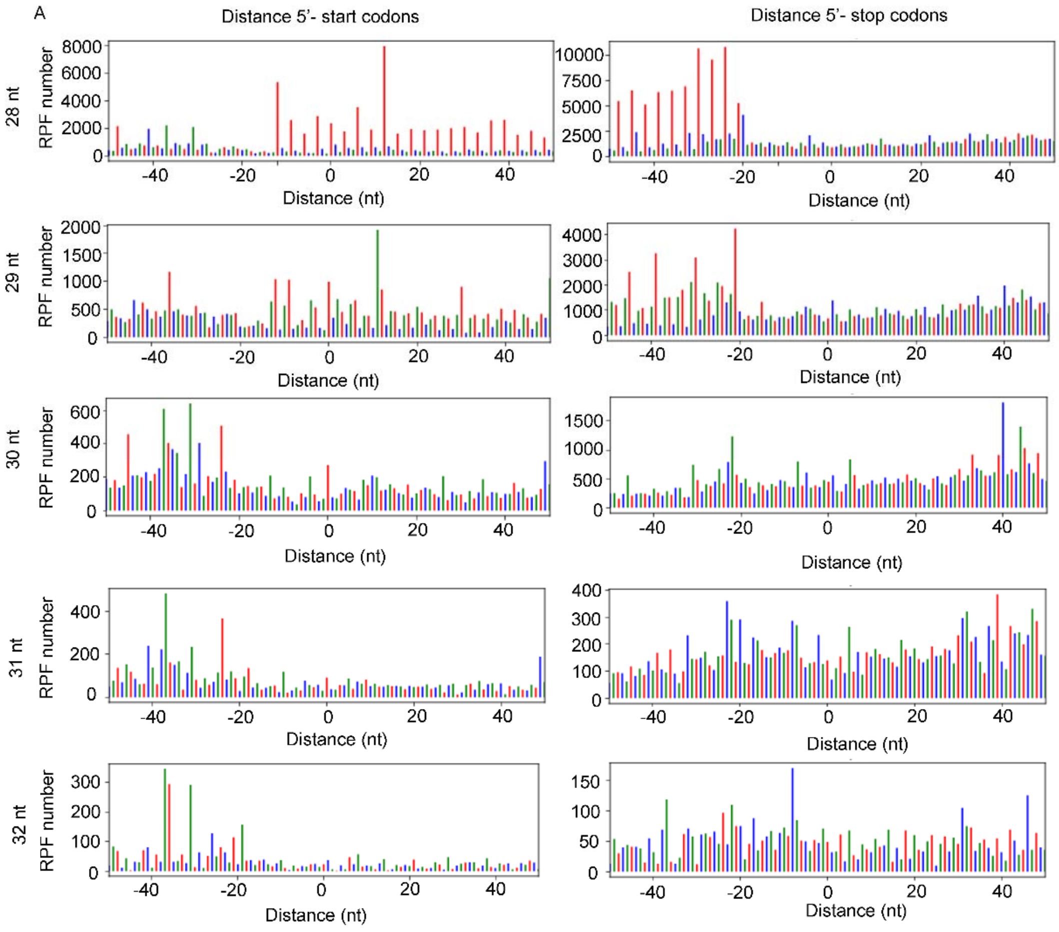

Supplementary Figure S2 | (A) Three-nucleotide periodicity in heathy seedlings.

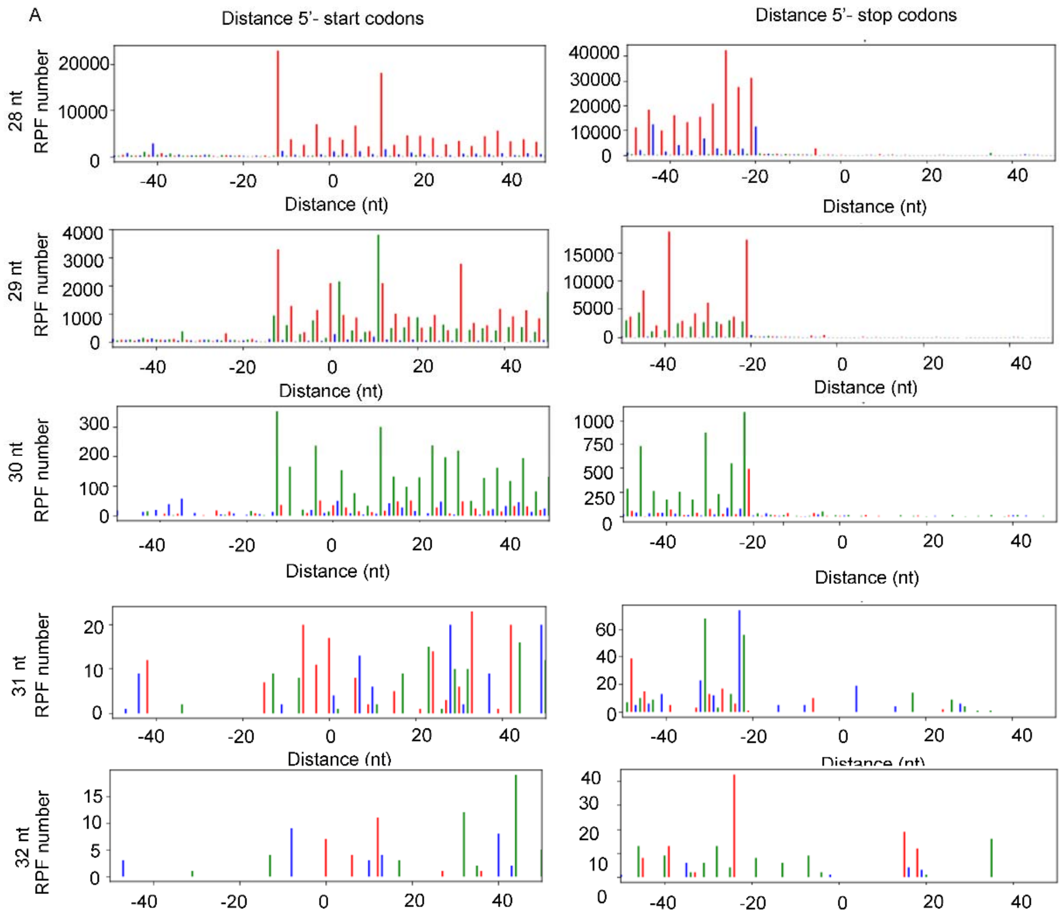

Supplementary Figure S3 | (A) Three-nucleotide periodicity in RSV-infected seedlings.
